# Serum Uric Acid Is a Weak Independent Predictor of Overall Survival in Older Adults

**DOI:** 10.3390/jcm10194505

**Published:** 2021-09-29

**Authors:** Mateusz Winder, Aleksander J. Owczarek, Małgorzata Mossakowska, Michał Holecki, Katarzyna Broczek, Tomasz Grodzicki, Tomasz Zdrojewski, Jerzy Chudek

**Affiliations:** 1 Department of Internal Medicine and Oncological Chemotherapy, Medical University of Silesia, 40-029 Katowice, Poland; chj@poczta.fm; 2 Department of Pathophysiology, Medical University of Silesia, 41-200 Katowice, Poland; aowczarek@paintbox.com.pl; 3 International Institute of Molecular and Cell Biology, 02-109 Warsaw, Poland; mmossakowska@iimcb.gov.pl; 4 Department of Internal, Autoimmune and Metabolic Diseases, Medical University of Silesia, 40-752 Katowice, Poland; holomed@gmail.com; 5 Department of Geriatrics, Medical University of Warsaw, 02-007 Warsaw, Poland; kbroczek@gmail.com; 6 Department of Internal Medicine and Gerontology, Jagiellonian University Medical College, 31-531 Krakow, Poland; tomekg@su.krakow.pl; 7 Department of Hypertension and Diabetology, Department of Preventive Medicine and Education, Medical University of Gdansk, 80-211 Gdansk, Poland; tomasz.zdrojewski@gumed.edu.pl

**Keywords:** serum uric acid, hyperuricemia, mortality, risk factor, old age, sex differences

## Abstract

Hyperuricemia accompanies many pathologies that contribute to overall death rate. The population-based multifaceted study of older adults in Poland made it possible to assess the effect of serum uric acid (SUA) on overall mortality. The PolSenior study performed between 2007–2011 included 3926 participants aged 65 years or above (mean age 79 ± 9 years) not treated with xanthin oxidase inhibitors (XOI) who were stratified by sex and SUA concentration into six subgroups increasing by 1 mg/dL. In 2019, survival data were retrieved from the population register. The crude risk of death was significantly higher in men and women with SUA ≥ 7 mg/dL. After adjustment to statistically significant factors, SUA remained a risk factor of death in men with SUA ≥ 8 mg/dL only, potentially due to the limited number of women with high SUA levels. Furthermore, age, heart failure, diabetes, and activities of daily living ≤ 4 pts were identified as factors increasing mortality risk regardless of sex. The risk of death increased also with smoking, past stroke, COPD/asthma, and hs-CRP > 3 mg/dL for men; and eGFR < 45 mL/min/1.73 m^2^, mini nutritional assessment ≤ 7 pts, and loop diuretics use for women. Mild hyperuricemia is a significant health status marker and an independent risk factor for overall mortality in older Caucasians not receiving XOI. Increased mortality is mostly limited to subjects with SUA levels ≥ 8 mg/dL.

## 1. Introduction

Uric acid (UA) has been a subject of medical interest for many years. The prevalence of hyperuricemia ranges between 5–16% in the general populations of Western Europe, reaching 85% of the Marshall Islands population [1,2]. High serum uric acid (SUA) concentration is linked with the development of gout; cardiovascular diseases (CVD), including coronary artery disease (CAD); stroke and hypertension; as well as diabetes and the accelerated progression of atherosclerosis [3,4,5,6,7,8,9]. Observational studies showed that both hyper- and hypouricemia are associated with increased prevalence of CVD-related and overall mortality [10,11].

Based on the UA solubility and the crystallization properties of monosodium urate (MSU), the upper limit for SUA ranges between 6–7 mg/dL, with reference values 1.5–6.0 mg/dL in women and 2.5–7.0 mg/dL in men [12,13]. Hyperuricemia is caused by an increased production and/or a reduced elimination of UA. Both these processes can be altered by the lifestyle (eating habits, alcohol intake, physical activity), comorbidities (obesity, metabolic syndrome, chronic kidney disease, myelo- and lymphoproliferative diseases) and medications (diuretics, aspirin, urate-lowering therapy (ULT), oncological treatment) [14]. SUA concentration is also determined by the expression of genes encoding UA transporters in the kidneys and intestine [15]. Variations of these genes in the form of risk alleles favor a higher rate of hyperuricemia and occur more frequently in Chinese, Japanese, Africans in Southwest USA, and Mexicans compared to Europeans [16]. Mendelian randomization studies demonstrate a correlation between xanthine oxidase (XO) genetic polymorphisms and SUA levels and a causal effect of SUA on the progression of related diseases [15,17]. Some scientific reports suggest that SUA may also be dependent on estrogen levels which down-regulate ABCG2 and SLC2A9 genes leading to decreased renal UA reabsorption [18]. This could explain the differences in the prevalence of hyperuricemia in men and women. SUA increases with age and the highest values are observed in older adults worldwide [19].

Hyperuricemia accompanies many pathologies that contribute to overall death rate; however, its impact on mortality is difficult to estimate. Studies on patients treated with allopurinol for gout and hyperuricemia showed inconsistent results regarding changes in all-cause and CVD-related mortality [20]. The link between SUA and CVD-related mortality in patients with acute myocardial infarction (MI) was reported in several studies [21,22,23]. A significantly elevated mortality risk has already been found in patients with SUA levels > 6.8 mg/dL [22,23]. However, studies evaluating the effect of SUA on mortality at levels above its upper limit of normal do not determine the exact concentration above which this effect is significant. Interestingly, an analysis based on the Framingham Heart Study showed no causal connection between SUA levels and incident coronary heart disease events, as well as CVD and all-cause mortality [24].

Kidneys are responsible for the elimination of circa 70% of UA, making glomerular filtration rate (GFR) the most important factor that determines SUA concentration in humans. Chronic kidney disease (CKD) with eGFR below 60 mL/min/1.73 m^2^ was shown to be a major risk factor in the development of hyperuricemia both in younger and older adults [12,14,25]. In addition, CKD is a significant risk factor for mortality in elderly resulting from CVD and end-stage renal diseases [26].

The Global Burden of Disease (GBD) study conducted in 2015 showed that global mortality caused by the diseases related to hyperuricemia especially diabetes and CKD increases worldwide [27]. Compared to GBD study from 2005, years of life lost (YLL) resulting from diabetes and CKD rose by 25.4% and 18.4%, respectively.

Metabolic syndrome (MetS) is another significant risk factor for all-cause and CVD-related mortality in general and older population [28]. Hyperuricemia is strongly associated with the occurrence of MetS components that attribute to the effects of insulin resistance (IR) on reduced urinary excretion of UA [29]. Recently, UA has been shown to participate in the development of insulin resistance by increasing mitochondrial oxidative stress [30].

It is largely unknown to what extent high SUA is an independent predictor of all-cause and CVD-related mortality in adults. This study aimed to analyze the importance of SUA levels as an independent predictor of mortality in older population using outcome data from the PolSenior study—a large, nation-wide project assessing health status of representative cohort of older adults in Poland. This comprehensive and multidisciplinary study was performed between 2007 and 2011, according to the protocol described previously in detail [31], and included 4979 subjects aged 65 years and over, with a large number of participants over 90 years old.

## 2. Patients and Methods

### 2.1. Study Population

**Figure 1:**
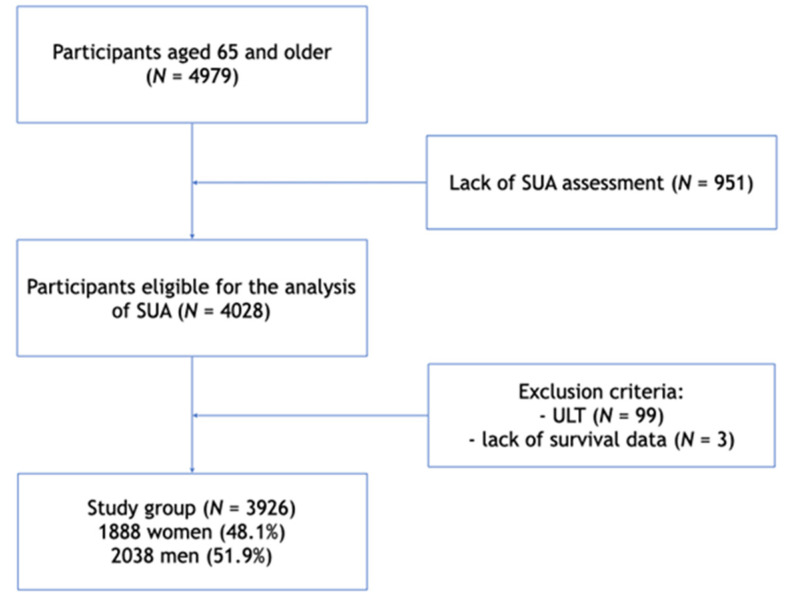
Flow chart. SUA—serum uric acid, ULT—urate-lowering therapy (treatment with xanthin oxidase inhibitors).

SUA assessment was a part of the PolSenior project. Prospective analysis of the overall survival included 3926 participants—1888 women (48.1%) and 2038 men (51.9%) with measured SUA levels, not receiving ULT (all xanthine oxidase inhibitors available on the polish market i.e., allopurinol and febuxostat) and with known survival time (Figure 1). Survival data of the study subjects were retrieved from the population register. The database access in 2019 indicated that 2214 (56.4%) of PolSenior subjects have died during 6.5 ± 3.3 years following participation in the study.

All assessments were carried out according to relevant guidelines and regulations in subjects who signed informed consent. The study protocol was approved by the Bioethics Committee of the Medical University of Silesia (KNW-6501-38/I/08).

All standards for biochemical measurements and comorbidities definitions have been described previously [12].

### 2.2. Data Analysis

Participants were divided by sex and into six groups on the basis of SUA concentration increasing by 1 mg, i.e., <4.0 mg/dL, 4.0–4.99 mg/dL, 5.0–5.99 mg/dL, 6.0–6.99 mg/dL, 7.0–7.99 mg/dL, and ≥8.0 mg/dL. The low prevalence of levels below 4 mg/dL and over 8 mg/dL precluded the analysis of additional subgroups. SUA concentration of 5.0–5.99 mg/dL (N = 1010) became the reference group, as it was characterized by the lowest death rate. The analysis used sociodemographic data which included sex, age, and elements of the comprehensive geriatric assessment. The latter includes activities of daily living (ADL, score 0–6) and mini-nutritional assessment; short form (MNA-SF, score 0–14) comorbidities, i.e., obesity with BMI and diabetes (DM), and hypertension with systolic and diastolic blood pressure (mean values); CKD based on the eGFR (full MDRD formula), CAD, past stroke, heart failure (HF), atrial fibrillation (AF), chronic obstructive pulmonary disease (COPD), or asthma; laboratory results, i.e., levels of total cholesterol (and hypercholesterolemia), triglycerides (and hypertriglyceridemia), or CRP (hs-CRP); medications, i.e., allopurinol, statins, and aspirin; and smoking and alcohol consumption.

### 2.3. Statistical Analysis

Statistical analyses were performed using STATISTICA 13.0 PL (TIBCO Software Inc., Palo Alto, CA, USA), StataSE 13.0 (StataCorp LP, TX, USA), and the R software (R Core Team (2013), R Foundation for Statistical ComputingVienna, Austria, http://www.R-project.org/ (accessed on 20 September 2021). Statistical significance was set at a *p*-value below 0.05. All tests were two-tailed. Imputations were not performed for missing data. Nominal and ordinal data were expressed as percentages. Interval data were expressed as the mean value ± standard deviation in the case of normal distribution. In the case of data with skewed or non-normal distribution, they were expressed as the median, with lower and upper quartiles. The distribution of variables was evaluated by the Anderson–Darling test and the quantile-quantile (Q-Q) plot. Homogeneity of variances was assessed by the Levene test. Nominal and ordinal data were compared with the χ^2^ test. Comparisons between groups for interval data were carried out with a one-way analysis of variances with Dunnett’s post-hoc test. Risk of death according to the SUA level was evaluated with the multivariable stepwise best logistic regression and shown with odds ratios (OR) and corresponding confidence intervals (±95% CI) and *p*-values. Overall mortality risk factors were assessed with a Pointwise Nonparametric Estimation of Hazard Ratio method (R package *smoothHR*). This method provides flexible hazard ratio curves allowing non-linear relationships between continuous predictors and survival, as well as a better understanding of the effects that each continuous covariate has on the outcome. Results are expressed in terms of hazard ratio curves, taking a specific covariate value as a reference. Results are presented with hazard ratios and graphically with smooth HR plots. The proportionality assumption were tested based on the Schoenfeld residuals (R function *cox.zph*). Multiple-collinearity was checked based on the correlation matrix of coefficients of the survival model. Additionally, overall survival analyses were performed with Kaplan–Meyer curves, stratified by SUA levels and sex, with the log-rank test to compare survival curves.

## 3. Results

### 3.1. Baseline Patient’s Characteristics

The detailed characteristics of the study groups are presented in Table 1A (men) and Table 1B (women).

The mean age of the analyzed group was 79 ± 9 years. The median SUA level was 5.6 (quartiles: 4.7–6.6) mg/dL for men and 5.2 (quartiles: 4.2–6.1) mg/dL for women. Subgroups with the highest SUA levels were significantly older, but the difference in age between extreme subgroups was much greater in women.

There was an increase in the prevalence of obesity, hypertension, HF, CAD, AF, CKD (defined as eGFR < 45 mL/min/1.73 m^2^), and hypertriglyceridemia, as well as the use of aspirin, statins, and all types of diuretics across consecutive SUA groups, both in men and women. Contrary, the percentage of malnourished (MNA ≤ 7 pts) was highest in subgroups with the lowest SUA levels and the highest percentage of individuals with ADL ≤ 4 pts was observed in subgroups with SUA < 4 mg/dL and ≥8 mg/dL. The association between ADL and SUA categories was U shaped.

### 3.2. Outcome Data

During the period of observation, i.e., 6.5 ± 3.3 years, 2214 (56.4%) of PolSenior participants died. The overall risk of death was greater among men (63.4%) (95% CI: 61.3–65.5%) than women (48.8%) (95% CI: 46.6–51.1%); *p* < 0.001. There was almost a linear increase in the frequency of death across the 5-year age groups: from 15.6% in the age of 65–69 to 86.3% in ≥85 years in women, and from 30.3% in the age of 65–69 to 92.2% in ≥85 years in men, estimated at a 15.6% and 18.6% increase in overall mortality per stratum in men and women, respectively.

**Figure 2:**
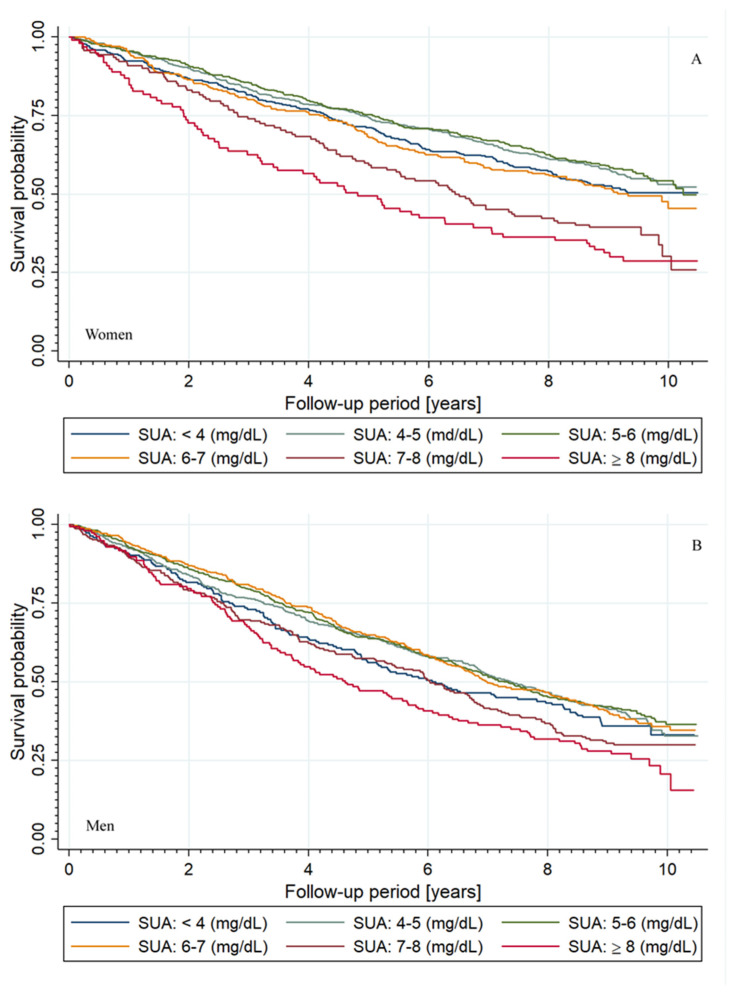
Kaplan–Meier survival estimates according to the SUA level subgroups. In comparison to the lowest cohort, groups with SUA ≥ 7 have increased overall mortality over time (*p* < 0.01). (**A**) Survival estimates for women; (**B**) survival estimates for men.

Kaplan–Meyer analysis revealed than both men and women with SUA levels equal to or greater than 7 mg/dL, in comparison to the lowest cohort, had increased overall mortality through time (*p* < 0.01)—Figure 2. There was no difference in mortality between men and women for cohorts with SUA levels between 7 and 8 mg/dL—*p* = 0.23 and ≥8 mg/dL—*p* = 0.72, in contrast to the cohorts with lower SUA levels (*p* < 0.001).

Subgroup analysis showed that higher SUA concentrations were associated with an increased prevalence of some of the investigated factors. The most prominent difference between groups with the lowest (<4 mg/dL) and the highest (≥8 mg/dL) SUA concentration concerned the incidence of low eGFR (<45 mL/min/1.73 m^2^), which increased from 1% to 43.2% in men and from 1.6% to 71.4%, in women. A statistically significant increase was also observed for BMI including BMI > 30, hypertension, CAD, HF, AF, and hypertriglyceridemia. DM prevalence increased significantly in women only (19.4% to 40.4%), whereas hypercholesterolemia increased significantly only in men (55.1% to 72.0%).

### 3.3. Non-Parametric Estimation of Hazard Ratio Curves

**Figure 3:**
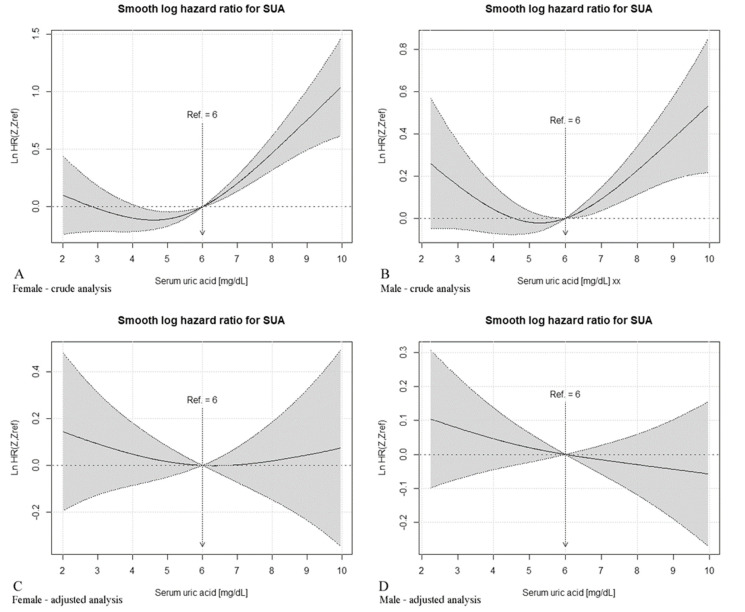
Adjusted and unadjusted nonparametric estimates of the dependence of all-time risk of death on SUA level without a prior diagnosis of diabetes mellitus among women and men (log hazard ratio, with 95% confidence limits). (**A**) The crude analysis for female; (**B**) The crude analysis for male; (**C**) The adjusted analysis for female; (**D**) The adjusted analysis for male.

In the univariable survival regression model, SUA was used as a continuous predictor. Results are presented in Figure 3. It can be observed that the hazard ratio of death increased almost linearly from value 6 mg/dL both for men and women in case of unadjusted analysis. As the confidence intervals in these range do not possess a 0 value, such a risk is statistically significant, in contrast to values below 6 mg/dL. Nevertheless, in adjusted analysis, no statistically significant risks were found regardless of the SUA concentration (the confidence intervals possess 0 value).

In the multivariable stepwise best survival regression model, the following factors were included: SUA as a continuous predictor; age; smoking; BMI including BMI > 30 kg/m^2^; past stroke; HF; AF; DM; eGFR < 45 mL/min/1.73 m^2^; COPD/asthma occurrence, serum levels of triglycerides, and hs-CRP > 3 mg/dL, ADL ≤ 4 pts, MNA ≤ 7 pts; as well as medications including statins, aspirin, hydrochlorothiazide, thiazide-like diuretics, loop-diuretics, and spironolactone.

Age, heart failure, diabetes, and ADL ≤ 4 pts proved to be the factors increasing mortality risk regardless of sex. The risk of death increased with smoking, past stroke, COPD/asthma occurrence, and hs-CRP > 3 mg/dL for men, and with eGFR < 45 mL/min/1.73 m^2^, MNA ≤ 7 pts, and loop diuretics use for women. The use of statins in women proved to be the only protective factor. Results are presented in Figure 3 and in Table 2 and Table 3.

**Figure 4:**
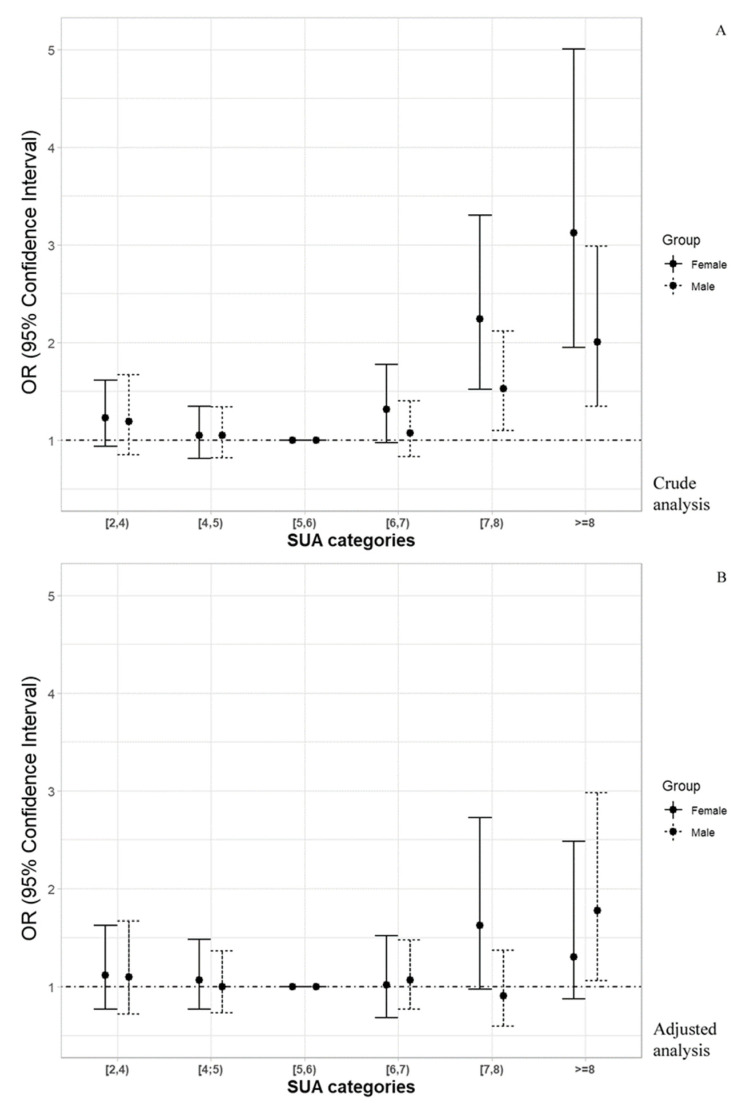
Risk of death (odds ratios with 95% confidence intervals) according to SUA levels subgroups. (**A**) The crude analysis; (**B**) The adjusted analysis.

To analyze the death occurrence, independently to survival time, the logistic regression was used. The univariable and multivariable stepwise best logistic regression with the above-mentioned factors were used to assess crude, as well as adjusted, death risk in SUA levels subgroups. Results displayed as odds ratios with corresponding 95% confidence intervals are presented in Table 4 as well as on the Figure 4.

The crude risk of death was significantly higher in the groups with SUA ≥ 7 mg/dL and the difference was more pronounced in women than in men. After adjustment to statistically significant factors, SUA remained a risk factor of death only in men with SUA ≥ 8 mg/dL, potentially due to small number of women with high SUA levels.

## 4. Discussion

The possible benefits of ULT have been explored over the past few decades. There is an ongoing discussion whether mild hyperuricemia is a risk indicator or an independent causal factor for increased cardiovascular morbidity and mortality, and whether asymptomatic, non-severe hyperuricemia should be treated [32]. To date, the role of such treatment in diseases other than hyperuricemia has not been established. There is still insufficient evidence to support urate lowering in patients with mild hyperuricemia that is not associated with gout, as ULT does not produce benefits on clinical outcomes including cardiovascular adverse events, all-cause mortality and kidney failure [33]. The results of the present study, even though not groundbreaking, add new information about the risk of death in an older population. We observed increased mortality in older Polish population with SUA levels greater than 8 mg/dL; however, this is more convincingly confirmed by the results of the presented analyzes in men.

We have identified several covariates that contribute to increased mortality in subjects both with low (4–5 mg/dL) or very low (<4 mg/dL) and increased (6–8 mg/dL) or very high (>8 mg/dL) SUA levels. The highest incidence of malnourishment (MNA ≤ 7 pts) and dependency in activities of daily living (ADL ≤ 4 pts) were characteristic for the group with the lowest SUA concentration. Similarly, a low ADL score was also observed in the group with the highest SUA concentration. The frequency of known risk factors explaining higher mortality in the elderly, such as age, HF, and DM, increased proportionally to SUA concentration in both sexes. Additionally, men had a higher mortality rate due to smoking, serum hs-CRP > 3 mg/dL, past stroke, and COPD/asthma occurrence, while women did due to CKD. Nevertheless, the adjusted risk of death turned out to be statistically significant solely in men when the SUA concentration was above 8 mg/dL. Interestingly, the use of statins was shown to reduce mortality in older women, only.

The incidence of CKD was found to correlate best with an increase in SUA levels and, therefore, it may be considered the main cause of hyperuricemia in older adults. CKD increases the risk of death from both renal and cardiovascular causes [26]. Thus, the association of higher mortality observed in individuals with high SUA concentrations and high prevalence of CKD is complex and strongly colinear. The association of high SUA levels and CKD is reciprocal. In some adults, the occurrence of high SUA level might precede the development of CKD by many years [34,35]. Urate nephropathy was very frequent in patients with goat in the past [36]; however, the epidemiology of urate nephropathy in the era of XO inhibitors is poorly known [37].

Elevated SUA is also observed in the course of HF. The complex pathogenesis of hyperuricemia in HF patients results from the coexistence of oxidative stress; decreased UA excretion, due to cardiorenal syndrome (CRS); comorbidities and the use of medications, such as loop and thiazide-like diuretics; and low doses of acetylsalicylic acid [38].

A similar correlation occurs with the components of MetS in which the IR-related hyperinsulinemia leads to a decreased UA elimination by the kidneys by enhancing the URAT1 gene expression and increasing the reabsorption of UA in the proximal tubule. Hypertension, hypertriglyceridemia, obesity, and low HDL and HF contribute to higher mortality [39,40]. In the present study, the incidence of hypertension, hypertriglyceridemia, obesity, and diabetes increased in each successive subgroup with higher SUA values, but only diabetes increased the risk of death.

SUA-related all-cause and cardiovascular mortality has been studied in other senescent populations. In a Japanese study [41], after the adjustment to age, hypertension, diabetes, obesity, hypercholesterolemia, smoking, alcohol consumption, eGFR, and proteinuria, SUA levels equal to or higher than 7 mg/dL proved to be an independent risk factor for all-cause and cardiovascular mortality in women.

A study performed on the Taiwanese population over 65 years old showed that SUA was an independent marker for all-cause and CVD-related mortality when its concentration was below 4 mg/dL and above 8 mg/dL [42]. Increased mortality in the group with low SUA concentrations particularly concerned people with malnutrition. Nutritional status analysis in our study showed that participants with MNA < 4 pts were prevalent in the subgroups with the lowest and highest SUA values, but mortality was related to malnutrition in women only. The Taiwanese study did not include the use of diuretics which in our study was associated with increased risk of death in women.

Results similar to Japanese and Taiwanese data were obtained by the authors of a study examining the effect of SUA on mortality in the Irish adult population (>18 years old) [43]. The pattern of SUA and all-cause mortality was U-shaped in men, and J-shaped in women, with the risk of mortality elevating significantly with SUA levels of <5.11 mg/dL and >7.63 mg/dL in men and exceeding 6.88 mg/dL in women.

The contrasting results of SUA-related mortality between the above mentioned and our study may result from racial and age differences of the examined populations. The highest prevalence of hyperuricemia was observed among the Taiwanese and Irish older populations [12,19]. The mean age of the participants also differed significantly, reaching 53.8 ± 15.5, 62.3 ± 10.4, and 72.6 ± 6.3 years in Irish, Japanese, and Taiwanese, respectively, as compared to 79 ± 9 years in the Polish population.

Contrary to the results of the previously cited studies [11,42,43], hypouricemia did not influence the adjusted risk of death in Polish older adults. The unadjusted risk of all-cause mortality did, however, show U-shape and J-shape pattern in men and women, respectively. Increased mortality rate in the group with the lowest SUA levels was explained by the highest incidence of malnutrition (11.4% and 21.1% in men and women, respectively) and a high incidence of dependency in activities of daily living, resulting in low ADL scores among these patients. When adjusted to significant covariates, the pattern was flattened and showed insignificantly weak correlation. Furthermore, the group with the lowest SUA values (<4 mg/dL) was characterized by the lowest incidence of obesity, hypertension, CAD, HF, AF, hypertriglyceridemia, and CKD, scored by eGFR values < 45 mL/min/1.73 m^2^, regardless of sex.

Other reports of studies conducted in the Japanese and US populations showed no relationship between SUA concentration and mortality in the general population [24,44]. It is worth noting that the participants of these studies were much younger, with mean age of 49.7 ± 13 and 49.9 ± 13.1 years for Japanese and 46 ± 15 and 48 ± 16 years for US men and women, respectively. In addition, the mean SUA concentrations were lower than those observed in older populations, especially in women.

The above-mentioned studies did not allow for a clear consensus regarding the impact of SUA on the risk of mortality, both in the general population and older adults. This is in line with the meta-analysis by Chan and colleagues, based on 28 clinical trials, in which the authors did not find the benefits of ULT in reducing major cardiovascular events, death, or kidney failure [33].

Nevertheless, we do believe that, beyond indisputable ULT of the gout, it seems reasonable to treat patients with severe asymptomatic hyperuricemia with coexisting cardiovascular comorbidities which are especially frequent in seniors. Having in mind racial discrepancies, our study provides arguments supporting the use of ULT in older Caucasian men with SUA > 8 mg/dL, which is in line with current guidelines [45,46]. We failed to show the cut-off value for women, which was probably related to the low percentage of women with SUA levels over 8 mg/dL—only 5.2% of the cohort. However, similar overall mortality (seen in Kaplan–Meyer curves) for men and women with SUA ≥ 7 mg/dL suggest that the indications for ULT should be similar for both sexes. Nonetheless, there are reports suggesting that consideration should be given to lowering the target SUA levels to below 6 mg/dL regardless of age and gender [47]. Due to the multifactorial nature of SUA concentration, the discussion on this subject would require additional research and appropriate analyzes in various population groups.

Our study had some limitations, such as a single SUA assessment (related to cross-sectional study design) and the lack of data concerning changes in pharmacotherapy, including eventual later implementation of ULT as well as the lack of dietary assessment in the whole study population, that precluded its implementation in the analysis. The exclusion of the 22% of the PolSenior study participants from this analysis was mostly due to the lack of blood withdrawal with a higher prevalence in the oldest. This might cause some sample distortion bias. We were also unable to identity the causes of death of the PolSenior participants due to limitations associated with the population register, i.e., how to perform sensitivity analysis for cardiovascular mortality. In addition, we carefully excluded covariates in the mortality risk adjustment, whose pathogenesis is significantly associated with hyperuricemia, including hypertension, CAD, and AF. This prevented over-adjustment for the main noncommunicable causes of death in the general population.

A comprehensive, multidisciplinary geriatric assessment, which included nutritional status, comorbidity, medications, blood tests, and a large older group, as well as the availability of complete long-term outcome data covering a period of 6.5 ± 3.3 years are the strengths of our study. We also assessed the impact of ADL—a rarely studied parameter that turned out to be an important prognostic factor for survival, which is an additional advantage of the study.

## 5. Conclusions

In summary, in our population-based cohort of older Caucasians, HF, diabetes, and ADL ≤ 4 pts are the factors which increase mortality risk regardless of sex. The risk of death also increased in men who were smokers, stroke survivors, suffering from COPD/asthma, and with hs-CRP > 3 mg/dL, and in women with eGFR < 45 mL/min/1.73 m^2^, MNA ≤ 7 pts, and prescribed with loop diuretics. A low ADL score (≤4) is observed in both groups with the lowest and the highest SUA concentrations. In addition, SUA levels equal to or greater than 8 mg/dL are increasing mortality; however, the analyses are convincing in men only.

Mild hyperuricemia is a significant health status marker and an independent risk factor for overall mortality in older Caucasians not receiving XOI. Increased mortality is mostly limited to subjects with SUA levels equal to or greater than 8 mg/dL.

## Figures and Tables

**Table 1:** (**A**). The characteristics of the male SUA level subgroups (*N* = 2038; 51.9%). (**B**) The characteristics of the female SUA level subgroups (*N* = 1888; 48.1%).

(A)							
SUA Level (mg/dL)	<4	<4;5)	<5;6)	<6;7)	<7;8)	≥8	*p*
N (%)	196 (9.6)	493 (24.2)	565 (27.7)	399 (19.6)	228 (11.2)	157 (7.7)	–
Death, N (%)	126 (64.3)	302 (61.3)	340 (60.2)	247 (61.9)	159 (69.7) *	118 (75.2) ^#^	<0.01
Age (years)	80 ± 9	79 ± 9	79 ± 9	78 ± 8	80 ± 9	81 ± 8 *	<0.05
SUA (mg/dL)	3.63 (3.29–3.81)	4.57 (4.29–4.76)	5.49 (5.22–5.72)	6.44 (6.19–6.73)	7.43 (7.21–7.70)	8.87 (8.24–9.69)	–
Smoking, *N* (%)	134 (68.4)	324 (65.7)	378 (66.9)	275 (68.9)	152 (66.7)	107 (68.2)	0.94
Alcohol consumption, *N* (%)	57 (29.5) ^#^	119 (24.5) *	108 (19.3)	96 (24.3)	50 (22.1)	28 (18.2)	<0.05
BMI (kg/m^2^)	25.9 ± 4.7 ^#^	26.0 ± 4.1 ^#^	27.4 ± 4.3	28.2 ± 4.2 *	28.6 ± 4.4 **	28.5 ± 4.7 *	<0.001
BMI > (30 kg/m^2^), *N* (%)	34 (18.7) *	70 (14.9) ^#^	138 (25.0)	113 (29.6)	79 (36.7) **	50 (33.1)	<0.001
Diabetes, *N* (%)	43 (21.9)	107 (21.7)	103 (18.3)	77 (19.4)	58 (25.6)	35 (22.3)	0.27
SBP (mm/Hg)	143 ± 21	144 ± 21	146 ± 21	145 ± 22	143 ± 24	142 ± 25	0.32
DBP (mmHg)	79 ± 11 *	81 ± 11	82 ± 11	82 ± 11	81 ± 12	80 ± 12	<0.05
Hypertension, *N* (%)	120 (61.2) *	303 (61.8) ^#^	394 (70.1)	290 (72.9)	167 (73.6)	122 (77.7) *	<0.001
Past stroke, *N* (%)	16 (8.2)	44 (8.9)	47 (8.4)	40 (10.1)	23 (10.1)	18 (11.5)	0.81
Coronary artery disease, *N* (%)	35 (17.9)	77 (15.6) ^#^	126 (22.3)	107 (26.8)	71 (31.1) **	54 (34.4) **	<0.001
Past myocardial infarction, *N* (%)	8 (4.1)	17 (3.4)	32 (5.7)	31 (7.8)	19 (8.3)	11 (7.0)	<0.05
Heart failure, *N* (%)	7 (3.7)	18 (3.8)	32 (5.8)	20 (5.1)	25 (11.2) **	30 (19.6) ^#^	<0.001
Atrial fibrillation, *N* (%)	23 (12.2)	63 (13.3)	96 (18.0)	55 (14.7)	53 (25.4) *	37 (25.7)	<0.001
Cholesterol (mg/dL)	184.1 ± 41.8 **	191.7 ± 42.5	195.9 ± 43.5	195.7 ± 44.2	187.6 ± 44.7	196.4 ± 49.8	<0.01
Hypercholesterolemia, *N* (%)	108 (55.1) ^#^	310 (62.9) *	390 (69.0)	299 (74.9) *	142 (62.3)	113 (72.0)	<0.001
Triglycerides (mg/dL)	90.1 ^#^ (69.0–116.1)	92.5 ^#^ (73.7–123.7)	104.6 (82.0–141.4)	117.8 ** (90.7–153.9)	108.8 (80.4–143.0)	131.7 ^#^ (102.3–172.1)	<0.001
Hypertriglyceridemia, *N* (%)	14 (7.1) ^#^	71 (14.4) *	114 (20.2)	114 (28.6) **	50 (21.9)	60 (38.2) ^#^	<0.001
eGFR (mL/min/1.73 m^2^) full	81.7 ± 16.5 ^#^	75.2 ± 15.8 ^#^	69.3 ± 15.4	64.3 ± 15.5 ^#^	57.5 ± 16.8 ^#^	48.9 ± 17.5 ^#^	<0.001
eGFR < 45 mL/min/1.73 m^2^, *N* (%)	2 (1.0) ^#^	8 (1.6) ^#^	25 (4.4)	44 (11.0) ^#^	53 (23.2) ^#^	67 (43.2) ^#^	<0.001
ACR (mg/g)	5.0 (2.1–16.4)	5.4 (1.8–16.0)	4.2 (1.8–17.2)	4.0 (1.5–12.1)	5.1 (1.9–23.8)	7.5 * (2.3–32.2)	<0.05
ACR ≥ 30 mg/g, *N* (%)	26 (14.4)	78 (17.0)	86 (15.9)	46 (12.4)	47 (22.0)	37 (25.2) *	<0.01
COPD/Asthma, *N* (%)	37 (19.1)	81 (16.5)	102 (18.1)	87 (21.9)	55 (24.3)	38 (24.4)	0.06
hs-CRP (mg/dL)	1.6 (0.8–4.1)	1.8 (0.8–4.1)	2.1 (1.0–4.5)	2.5 (1.3–5.5)	3.3 ^#^ (1.6–6.0)	2.9 ^#^ (1.7–6.7)	<0.001
CRP > 3 mg/dL, *N* (%)	65 (33.5)	178 (36.2)	207 (36.8)	176 (44.4) *	121 (53.8) ^#^	75 (48.1) *	<0.001
ADL ≤ 4 pts, *N* (%)	26 (13.3) **	52 (10.6) *	40 (7.1)	31 (7.8)	33 (14.5) **	21 (13.5) *	<0.01
MNA 8-11 pts (at risk), *N* (%)	79 (44.9)	176 (38.7)	209 (38.8)	118 (32.0)	79 (37.6)	56 (38.4)	<0.05
MNA ≤ 7 pts (malnourished), *N* (%)	20 (11.4)	44 (9.7)	36 (6.7)	27 (7.3)	17 (8.1)	14 (9.6)	
MetS, *N* (%)	66 (34.2) **	168 (34.4) ^#^	264 (47.1)	223 (56.3) **	132 (58.1) **	110 (70.5) ^#^	<0.001
Aspirin, *N* (%)	66 (33.7)	146 (29.6)	170 (30.1)	155 (38.8) **	102 (44.7) ^#^	60 (38.2) *	<0.001
Hydrochlorothiazide, *N* (%)	2 (1.0)	15 (3.0)	24 (4.3)	18 (4.5)	30 (13.2) ^#^	18 (11.5) ^#^	<0.001
Thiazide-like diuretics, *N* (%)	9 (4.6) *	28 (5.7)**	58 (10.3)	68 (17.0) **	46 (20.2) ^#^	41 (26.1) ^#^	<0.001
Loop diuretics, *N* (%)	5 (2.6)	18 (3.7)	30 (5.3)	32 (8.0)	40 (17.5) ^#^	53 (33.8) ^#^	<0.001
Spironolactone, *N* (%)	12 (6.1)	33 (6.7)	53 (9.4)	41 (10.3)	40 (17.5) **	38 (24.2) ^#^	<0.001
Diuretics, *N* (%)	22 (11.2) ^#^	73 (14.8) ^#^	131 (23.2)	130 (32.6) **	121 (53.1) ^#^	108 (68.8) ^#^	<0.001
Statins, *N* (%)	39 (19.9)	83 (16.8) *	128 (22.7)	111 (27.8)	59 (25.9)	43 (27.4)	<0.01
Fibrates, *N* (%)	1 (0.5)	8 (1.6)	2 (0.3)	7 (1.7)	2 (0.9)	1 (0.6)	0.21
Non-dihydropyridine calcium channel blockers, *N* (%)	5 (2.6)	23 (4.7)	24 (4.2)	21 (5.3)	9 (3.9)	10 (6.4)	0.58
Losartan, *N* (%)	10 (13.9)	8 (1.6)	19 (3.4)	17 (4.3)	10 (4.3)	8 (5.1)	0.11
Metformin, *N* (%)	6 (3.1)	9 (1.8)	23 (4.1)	18 (4.5)	8 (3.5)	6 (3.8)	0.30
**(B)**							
**SUA Level**	**<4**	**<4;5)**	**<5;6)**	**<6;7)**	**<7;8)**	**≥8**	***p***
*N* (%)	386 (20.5)	544 (28.8)	445 (23.6)	272 (14.4)	142 (7.5)	99 (5.2)	–
Death, *N* (%)	188 (48.7)	243 (44.7)	194 (43.6)	137 (50.4)	90 (63.4) ^#^	70 (70.7) ^#^	<0.001
Age (years)	78 ± 9	78 ± 9	78 ± 8	79 ± 9	81 ± 9 **	84 ± 8 ^#^	<0.001
SUA (mg/dL)	3.48 (3.11–3.72)	4.52 (4.29–4.76)	5.44 (5.23–5.69)	6.43 (6.19–6.63)	7.37 (7.16–7.61)	8.62 (8.23–9.50)	–
Smoking, *N* (%)	63 (16.3)	109 (20.0)	83 (18.7)	57 (21.0)	22 (15.5)	14 (14.1)	0.38
Alcohol consumption, *N* (%)	186 (48.8)	263 (49.6)	225 (51.5)	133 (50.8)	73 (52.1)	56 (56.6)	0.78
BMI (kg/m^2^)	26.3 ± 4.9 ^#^	28.3 ± 4.9 ^#^	29.8 ± 5.1	31 ± 5.6 *	30.9 ± 6.1	30.4 ± 6.2	<0.001
BMI (kg/m^2^) > 30, *N* (%)	79 (22.6) ^#^	176 (34.2) **	185 (43.5)	141 (55.3) **	68 (51.9)	45 (49.5)	<0.001
Diabetes, N (%)	75 (19.4)	102 (18.8)	99 (22.2)	91 (33.6) **	51 (35.9) **	40 (40.4) ^#^	<0.001
SBP (mm/Hg)	144 ± 22	146 ± 22	146 ± 21	146 ± 22	126	142 ± 25	0.29
DBP (mmHg)	83 ± 11 *	85 ± 11	85 ± 11	86 ± 11	87 ± 11	84 ± 12	<0.05
Hypertension, *N* (%)	258 (67.4) ^#^	425 (78.1)	353 (80)	224 (82.7)	126 (89.4) *	80 (80.8)	<0.001
Past stroke, *N* (%)	25 (6.5)	33 (6.1)	33 (7.4)	18 (6.6)	19 (13.4) *	14 (14.3) *	<0.01
Coronary artery disease, *N* (%)	56 (14.5) *	81 (14.9) *	92 (20.7)	61 (22.4)	44 (31.0) *	30 (30.3) *	<0.001
Past myocardial infarction, *N* (%)	8 (2.1)	16 (2.9)	8 (1.8)	8 (2.9)	8 (5.6)	8 (8.1)	<0.01
Heart failure, *N* (%)	14 (3.7)	19 (3.6)	19 (4.4)	14 (5.2)	16 (11.7) **	18 (18.9) ^#^	<0.001
Atrial fibrillation, *N* (%)	62 (17.0)	88 (17.2)	83 (20.0)	56 (21.5)	37 (28.9)	24 (27.3)	<0.05
Cholesterol (mg/dL]	209.3 ± 44.1	211.8 ± 44.8	212.8 ± 44.3	211.2 ± 51.9	201 ± 47.3	206.6 ± 55.2	0.13
Hypercholesterolemia, *N* (%)	299 (77.5)	451 (82.9)	378 (84.9)	216 (79.4)	110 (77.5)	80 (80.8)	0.06
Triglycerides (mg/dL)	98.6 ^#^ (80.3–127.5)	116.3 ** (89.2–148.9)	126.6 (100.0–162.3)	136.7 * (106.5–177.4)	142.9 ^#^ (111.3–194.1)	142.1 ^#^ (119.5–193.0)	<0.001
Hypertriglyceridemia, *N* (%)	66 (17.1) ^#^	139 (25.6) **	152 (34.2)	111 (40.8)	68 (47.9) *	44 (44.4) *	<0.001
eGFR (mL/min/1.73 m^2^)	75.1 ± 16.5 ^#^	68.4 ± 14.8 ^#^	63.2 ± 14.1	56.4 ± 15.8 ^#^	48.9 ± 15.3 ^#^	39.6 ± 14.1 ^#^	<0.001
eGFR < 45 mL/min/1.73 m^2^, *N* (%)	6 (1.6) ^#^	19 (3.5) ^#^	40 (9.0)	64 (23.6) ^#^	58 (41.4) ^#^	70 (71.4) ^#^	<0.001
ACR (mg/g)	6.2 (2.3–15.9)	5.7 (2.3–14.8)	5.3 (2.2–15.6)	5.2 (2.2–16.6)	5.7 (2.5–21.4)	7.9 (2.6–38.1)	0.24
ACR ≥ 30 mg/g, *N* (%)	50 (14.5)	71 (14.4)	59 (14.1)	36 (14.2)	28 (21.5)	25 (27.5) **	<0.01
COPD/Astma, *N* (%)	60 (15.5)	76 (14)	70 (15.8)	49 (18.1)	22 (15.5)	26 (26.3)	0.07
hs-CRP (mg/dL)	2.0 * (0.9–3.9)	2.1 (1.1–4.3)	2.5 (1.3–4.5)	3.3 ^#^ (1.7–7.1)	3.7 ^#^ (1.7–6.7)	3.6 ** (1.7–7.9)	<0.001
hs-CRP > 3 mg/dL, *N* (%)	133 (34.5)	196 (36.2)	177 (40.0)	151 (56.1) ^#^	79 (55.6) ^#^	55 (56.7) **	<0.001
ADL ≤ 4 pts, *N* (%)	60 (15.6) **	55 (10.2)	40 (9.0)	38 (14.0) *	26 (18.3) **	22 (22.4) ^#^	<0.001
MNA 8–11 pts, *N* (%)	165 (48.4)	226 (45)	183 (43.9)	110 (43.5)	63 (49.2)	47 (54.0)	<0.001
MNA ≤ 7 pts, *N* (%)	72 (21.1)	66 (13.1)	45 (10.8)	29 (11.5)	16 (12.5)	16 (18.4)	
MetS, *N* (%)	160 (42.1) ^#^	307 (56.6) **	289 (65.7)	207 (76.4) **	116 (82.3) ^#^	78 (79.6) **	<0.001
Aspirin, *N* (%)	109 (28.2) *	176 (32.4)	159 (35.7)	83 (30.5)	54 (38.0)	43 (43.4)	<0.05
Hydrochlorothiazide, *N* (%)	9 (2.3) ^#^	29 (5.3)	34 (7.6)	26 (9.6)	29 (12.7) ^#^	15 (15.2) *	<0.001
Thiazide-like diuretics, *N* (%)	31 (8.0) ^#^	90 (14.7)	95 (21.3)	81 (29.8) *	45 (31.7) ^#^	26 (26.3)	<0.001
Loop diuretics, *N* (%)	9 (2.3) **	23 (4.2)	31 (7.0)	36 (13.2) **	29 (20.4) ^#^	44 (44.4) ^#^	<0.001
Spironolactone, *N* (%)	21 (5.4) **	52 (9.6)	50 (11.2)	51 (18.8) **	29 (20.4) **	33 (33.3) ^#^	<0.001
Diuretics, *N* (%)	62 (16.1) ^#^	151 (27.8) ^#^	174 (39.1)	152 (55.9) ^#^	97 (68.3) ^#^	83 (83.8) ^#^	<0.001
Statins, *N* (%)	71 (18.4) **	136 (25.0)	122 (27.4)	70 (25.7)	46 (32.4)	27 (27.3)	<0.05
Fibrates, *N* (%)	9 (2.3)	7 (1.3)	3 (0.7)	2 (0.7)	1 (0.7)	0	0.19
Non-dihydropyridine calcium channel blockers, *N* (%)	16 (4.1)	34 (6.2)	19 (4.3)	14 (5.1)	11 (7.7)	3 (3.0)	0.33
Losartan, *N* (%)	19 (4.9)	32 (5.9)	32 (7.2)	21 (7.7)	6 (4.2)	3 (3.0)	0.34
Metformin, *N* (%)	14 (3.6)	17 (3.1)	26 (5.8)	25 (9.2)	12 (8.4)	7 (7.1)	<0.01

* *p* < 0.05; ** *p* < 0.01; ^#^ *p* < 0.001. ACR—albumin to creatinine ratio, ADL—activity of daily living, BMI—body mass index, COPD—chronic obstructive pulmonary disease, DBP—diastolic blood pressure, eGFR—estimated glomerular filtration rate, hs-CRP—high sensitivity C-reactive protein, MetS—metabolic syndrome, MNA—mini nutritional assessment, SBP—systolic blood pressure, SUA—serum uric acid.

**Table 2:** Factors affecting the overall survival. Results of the univariate logistic regression analysis.

	Men		Women	
Factor	HR	±95% CI	HR	±95% CI
SUA (mg/dL)	1.05 *	1.01–1.09	1.12 ^#^	1.07–1.17
Age (per 10 years)	2.49 ^#^	2.32–2.67	3.44 ^#^	3.16–3.74
Smoking	1.13	0.99–1.27	1.55 ^#^	1.30–1.89
Past stroke	1.81 ^#^	1.52–2.15	1.89 ^#^	1.52–2.33
Heart failure	3.43 ^#^	2.82–4.16	4.24 ^#^	3.37–5.32
COPD/Asthma	1.63 ^#^	1.19–1.56	1.11	0.93–1.32
Diabetes	1.08	0.95–1.24	1.20 *	1.03–1.39
hs-CRP > 3 mg/dL	1.87 ^#^	1.68–2.09	1.30 ^#^	1.14–1.48
eGFR < 45 mL/min/1.73 m^2^	2.06 ^#^	1.74–2.43	2.54 ^#^	2.16–2.99
ADL ≤ 4 pts	4.25 ^#^	3.63–4.97	5.41 ^#^	4.62–6.32
MNA ≤ 7 pts	2.56 ^#^	2.14–3.06	2.55 ^#^	2.15–3.03
Statins	0.81 **	0.70–0.92	0.60 ^#^	0.51–0.71
Loop diuretics	1.20 **	1.06–1.35	1.24 **	1.09–1.42

* *p* < 0.05; ** *p* < 0.01; ^#^ *p* < 0.001. ADL—activities of daily living, COPD—chronic obstructive pulmonary disease, eGFR—estimated glomerular filtration rate, hs-CRP—high sensitivity C-reactive protein, MNA—mini-nutritional assessment, SUA—serum uric acid.

**Table 3:** Factors associated with overall mortality in non-parametric estimation of hazard ratio curves.

	Men		Women	
Factor	HR	±95% CI	HR	±95% CI
Age (per 10 years)	2.40 ^#^	2.20–2.57	2.91 ^#^	2.64–3.25
Smoking	1.42 ^#^	1.25–1.61	-	-
Past stroke	1.37 **	1.14–1.65	-	-
Heart failure	1.97 ^#^	1.60–2.43	2.23 ^#^	1.71–2.92
COPD/Asthma	1.15 *	1.00–1.32	-	-
Diabetes	1.32 ^#^	1.14–1.52	1.22 *	1.03–1.45
hs-CRP > 3 mg/dL	1.63 ^#^	1.45–1.83	-	-
eGFR < 45 mL/min/1.73 m^2^	-	-	1.32 *	1.06–1.64
ADL ≤ 4 pts	2.04 ^#^	1.70–2.44	1.60 ^#^	1.29–1.99
MNA ≤ 7 pts	-	-	1.38 **	1.14–1.66
Statins	-	-	0.81 *	0.67–0.98
Loop diuretics	-	-	1.43 **	1.14–1.80

* *p* < 0.05; ** *p* < 0.01; ^#^ *p* < 0.001. ADL—activities of daily living, COPD—chronic obstructive pulmonary disease, eGFR—estimated glomerular filtration rate, hs-CRP—high sensitivity C-reactive protein, MNA—mini-nutritional assessment, SUA—serum uric acid.

**Table 4:** The multivariable logistic regression analysis of the death risk in men (*N* = 2038; 51.9%) and women (*N* = 1888; 48.1%) SUA level subgroups.

SUA Level	<4	<4;5)	<5;6)	<6;7)	<7;8)	≥8	*p*
**Men**							
Death risk (OR (±95% CI))	1.191 (0.850–1.669)	1.046 (0.817–1.340)	Ref.	1.075 (0.827–1.399)	1.525 * (1.097–2.119)	2.002 ** (1.343–2.985)	-
Adjusted Death risk (OR (±95% CI)) ^a^	1.098 (0.721–1.672)	0.997 (0.728–1.366)	Ref.	1.064 (0.766–1.477)	0.905 (0.596–1.372)	1.777 * (1.060–2.980)	-
**Women**							
Death risk (OR (±95% CI))	1.228 (0.934–1.615)	1.045 (0.811–1.344)	Ref.	1.313 (0.970–1.777)	2.239 ^#^ (1.517–3.306)	3.123 ^#^ (1.949–5.005)	-
Adjusted Death risk (OR (±95% CI)) ^b^	1.118 (0.768–1.625)	1.066 (0.766–1.485)	Ref.	1.019 (0.683–1.520)	1.627 (0.972–2.725)	1.304 (0.840–2.484)	-

* *p* < 0.05; ** *p* < 0.01; ^a^ adjusted to age, smoking, past stroke, diabetes, heart failure, statins, COPD/Asthma, levels of cholesterol and CRP > 3 mg/dL and ADL ≤ 4 pts. ^b^ adjusted to age, heart failure, loop diuretics, ADL ≤ 4 pts and MNA ≤ 7 pts.

## Data Availability

The data presented in this study are available on request from the corresponding author.
